# 3D Atrial Strain for Predicting Recurrence of Atrial Fibrillation after Pulmonary Vein Isolation

**DOI:** 10.3390/jcm12113696

**Published:** 2023-05-26

**Authors:** Iva Krizanovic-Grgic, Shehab Anwer, Jan Steffel, Daniel Hofer, Ardan M. Saguner, Christina M. Spengler, Alexander Breitenstein, Felix C. Tanner

**Affiliations:** 1 Department of Cardiology, University Heart Center, University Hospital Zurich, 8091 Zurich, Switzerland; iva.krizanovic-grgic@usz.ch (I.K.-G.); alexander.breitenstein@usz.ch (A.B.); 2 Exercise Physiology Laboratory, Institute of Human Movement Sciences and Sport, Swiss Federal Institute of Technology in Zurich (ETH Zürich), 8092 Zurich, Switzerland; 3 Zurich Center for Integrative Human Physiology, University of Zurich, 8006 Zurich, Switzerland

**Keywords:** atrial fibrillation, atrial fibrillation recurrence, pulmonary vein isolation, 3D speckle-tracking echocardiography, 2D speckle-tracking echocardiography, 3D voltage map

## Abstract

Aims: Association of two-(2D) and three-dimensional (3D) left atrial strain (LAS) and low-voltage area (LVA) with recurrence of atrial fibrillation (AF) after pulmonary vein isolation (PVI) was assessed. Methods and results: 3D LAS, 2D LAS, and LVA were obtained in 93 consecutive patients undergoing PVI and recurrence of AF was analyzed prospectively. AF recurred in 12 patients (13%). The 3D left atrial reservoir strain (LARS) and pump strain (LAPS) were lower in patients with recurrent AF than without (*p* = 0.008 and *p* = 0.009, respectively). In univariable Cox regression, 3D LARS or LAPS were associated with recurrent AF (LARS: HR = 0.89 (0.81–0.99), *p* = 0.025; LAPS: HR = 1.40 (1.02–1.92), *p* = 0.040), while other values were not. Association of 3D LARS or LAPS with recurrent AF was independent of age, body mass index, arterial hypertension, left ventricular ejection fraction, and end-diastolic volume index and left atrial volume index in multivariable models. Kaplan–Meier curves revealed that patients with 3D LAPS < −5.9% did not exhibit recurrent AF, while those >−5.9% had a significant risk of recurrent AF. Conclusions: 3D LARS and LAPS were associated with recurrent AF after PVI. Association of 3D LAS was independent of relevant clinical and echocardiographic parameters and improved their predictive value. Hence, they may be applied for outcome prediction in patients undergoing PVI.

## 1. Introduction

Atrial fibrillation (AF) is one of the most common supraventricular arrhythmias affecting people worldwide. Due to demographic changes, the number of patients suffering AF will increase dramatically over the coming years. AF does not only cause symptoms such as palpitations but is associated with impaired survival and thromboembolic complications [1,2,3]. During the early stages of AF development, pulmonary veins represent the structural source for AF triggers. Hence, the primary goal of interventional catheter-guided ablation is to isolate these veins (PVI). This intervention is a safe and successful procedure; however, AF recurs relatively often with an incidence of 11% to 41% within the first year [1,4]. Several ablations may thus be needed in some patients to permanently isolate the pulmonary veins, carrying additional procedural risk and increasing health care cost. Therefore, it is important to identify predictors allowing to differentiate responders from non-responders.

During catheter intervention, a voltage map of the left atrium (LA) may be created for visualizing healthy and diseased areas of the left atrial wall. The latter are recognized by low-voltage regions representing fibrosis and/or scar tissue and have been demonstrated to predict AF recurrence [5].

Usually, patients undergo an echocardiographic examination before PVI to determine cardiac function and atrial size. Speckle-tracking echocardiography is suitable for analyzing deformation of all cardiac cavities, including the atria. Several studies showed that two-dimensional (2D) speckle tracking echocardiography might be useful for identification of responders and non-responders after PVI [6,7,8,9,10,11,12,13].

Since analysis of three-dimensional (3D) LA deformation has been improved lately using novel strain parameters revealing impaired atrial function before anatomical alterations are detectable [14], we aimed to explore whether (a) 3D LA strain (LAS) is associated with AF recurrence after PVI, (b) 2D or 3D LAS shows a stronger association with AF recurrence, and (c) how LAS methods compare to low voltage areas (LVA) with regard to association with AF recurrence.

## 2. Methods

### Study Population

**Figure 1:**
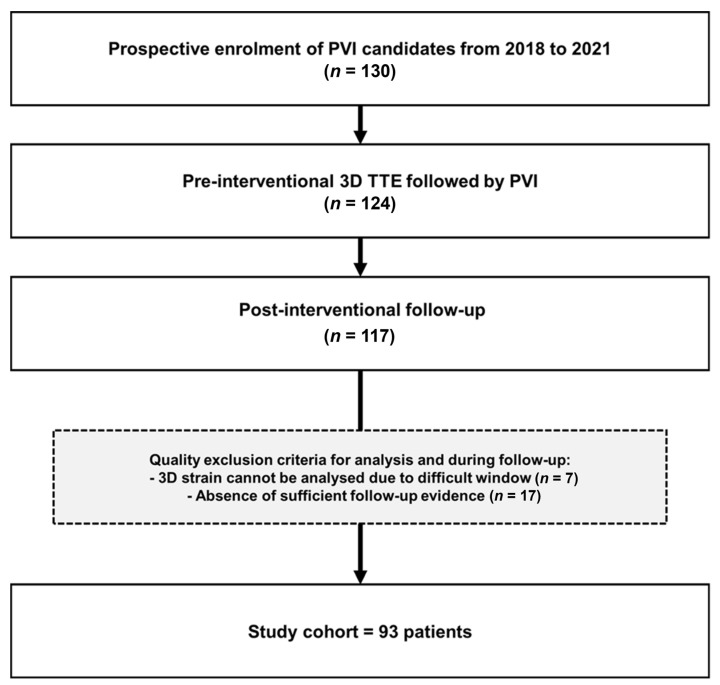
Study flow chart. 3D, three-dimensional; TTE, transthoracic echocardiography; PVI, pulmonary vein isolation.

This prospective single-center study enrolled 130 consecutive patients with AF undergoing radiofrequency ablation (RFA) for PVI between December 2018 and October 2021. The protocol was approved by the Ethics Committee Zürich (KEK-ZH-No. 2017–00737) and written informed consent for study participation was obtained from all patients. According to pre-specified criteria, all patients underwent a complete echocardiographic examination including 3D LAS one day prior to RFA or at the day of RFA. Among those scanned at the day of RFA, 89 out of 93 (95%) patients received the echocardiogram before the RFA and 4 out of 93 (5%) patients immediately after RFA. A 3D electro-anatomical voltage map (EAVM) was acquired during PVI. Inclusion criteria are provided in Figure 1. The final study population for 3D LAS and 3D EAVM consisted of 93 patients. The 2D LAS cohort was reduced to 87 patients due to technical problems with offline analysis. According to recurrence of AF during follow-up, patients were dichotomized into two groups: (a) Non-AF-Group and (b) AF-group. None of the patients exhibited a persistent supraventricular arrhythmia before the ablation procedure.

## 3. Echocardiography

**Figure 2:**
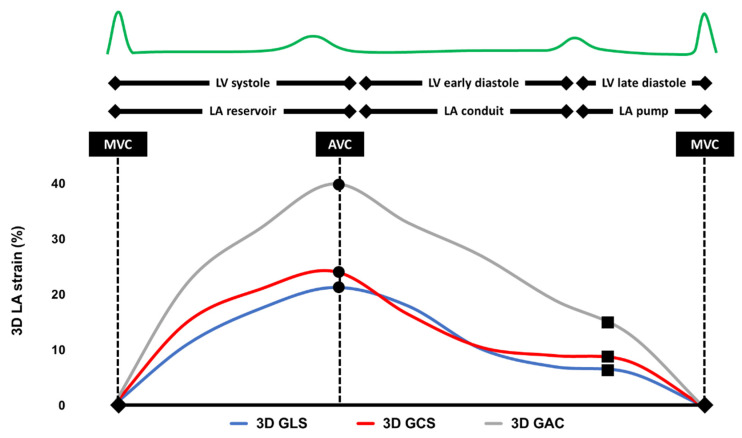
Typical example of a three-dimensional left atrial strain analysis. LV, left ventricle; LA, left atrium; MVC, mitral valve closure; AVC, aortic valve closure; 3D, three-dimensional; GLS, global longitudinal strain; GCS, global circumferential strain; and GAC, global area change.

All transthoracic echocardiographic (TTE) examinations were performed by experienced certified staff according to current recommendations using Canon Aplio i900 (Canon Medical Systems, Tokyo, Japan) [15]. All patients were in sinus rhythm (SR) during the echocardiographic examination. All 2D strain analyses were carried out by an experienced investigator using the atrial strain module in TomTec Image Arena Cardiac Performance Analysis (v.4.6) on images recorded, saved, and analyzed at a frame rate of >60 frames/s. All 3D strain analyses were carried out on the Canon Aplio i900 unit by the same investigator. Global 3D LAS was obtained according to current recommendations [16]. All 3D LA volumes were displayed in reconstructed apical four-chamber, apical two-chamber, and short-axis views and 3D cineloops were analyzed at 20–30 frames/s using the vendor software. To obtain correct 3D wall motion tracking the endocardial border was traced with the start point at the level of the mitral annulus in a counterclockwise direction. Pulmonary veins and left atrial appendage were excluded from tracing. The 3D wall motion tracking was automatically performed using a 3 mm region of interest (ROI) and manual corrections were applied to all the trackings as required during the entire cardiac cycle (Figure 2). The baseline of the deformation curve was set at end-diastole, as recommended [17]. If necessary, manual adjustments were made to provide the best possible quality. The deformation curves allowed to extract all 2D and 3D LAS parameters of interest, such as 2D biplane and 3D LA reservoir strain (LARS), as well as 2D biplane and 3D pump strain (LAPS), respectively.

Intra- and inter-observer agreement for 3D strain analyses were determined on 10 randomly selected echocardiographic studies. The correlation coefficient for 3D LA GLS intra-observer variability was r = 0.94 (*p* < 0.001) and for inter-observer variability r = 0.86 (*p* < 0.001), demonstrating strong inter- and intra-observer agreement.

## 4. Pulmonary Vein Ablation and Voltage Mapping

All RFA procedures were performed by four experienced operators according to current clinical practice. General anesthesia was used in all patients. Procedures were supported by the Carto 3 system (Biosense Webster, Irvine, CA, USA, Version V6–V7). Circumferential ablation around both ipsilateral PV was performed using either an open-irrigated ablation catheter (Thermocool SmartTouch^®^, Biosense Webster) or the HELIOSTAR™ Ballon Ablation catheter (Biosense Webster). The LA was mapped during SR after PVI and the EAVM were obtained using high-density mapping catheters (PentaRay^®^ or Lasso^®^, both from Biosense Webster). Only EAVM with at least 300 voltage points were considered for this study. LVA were denoted if the bipolar electrogram amplitude was <0.5 mV covering a minimal area of 1 square centimeter (cm^2^) of the total LA surface [18,19]. The interpolation and color threshold of EAVM were set to 15 mm [20]. LVA was measured once in cm^2^ and once in percentage (%) with the area measurement tool of the Carto 3 system (Biosense Webster).

## 5. Follow-Up and Endpoints

Follow-up started immediately after the intervention. The patients were followed-up by their treating cardiologist. Median follow-up time was 95 [IQR 83–110] days (mean = 110 days). Consistent with previous data, the blanking period was defined from 0 to 60 days after PVI [21,22,23,24]. Recurrences happening during this time period were not considered as failure of treatment. The endpoint was AF recurrence defined as AF, atrial tachycardia, or atrial flutter for a duration >30 s documented on an ECG after the blanking period. For ECG documentation either (1) 24 h Holter recording, (2) Apple Watch recording, or (3) cardiac device interrogation was used. Follow-up data were collected until the end of February 2022.

## 6. Statistical Analysis

All statistical analyses were performed using MedCalc^®^ for Windows Vista/7/8/10 (Version 19.6.4, MedCalc Software, Ostend, Belgium). Continuous variables were shown as median and interquartile ranges (IQR), while categorical variables were presented as numbers and percentages. Normality distribution was tested using the Shapiro–Wilk test. The Mann–Whitney U test was used for pairwise comparisons. The association of clinical, echocardiographic, or EAVM parameters with events during follow-up were tested using logistic and Cox regression analyses. Exploration of incremental value was tested by multivariable logistic regression. To report model fitness Chi-square log likelihood ratio (χ^2^) was used in comparison to the univariable model. Analyses were considered significant if the two-sided *p*-value was <0.05. ROC-curves were generated to determine cut-off values. The Kaplan–Meier method was used to analyze cumulative event rates. Kaplan–Meier curves were truncated after 6 months due to censoring of a high patient number.

## 7. Results

### 7.1. Baseline Characteristics

Baseline characteristics of the study population are summarized in Table 1. Mean age was 63.9 years, 25% were females, and the majority of participants suffered from arterial hypertension. There were no significant differences in baseline parameters between patients with and without recurrent AF.

### 7.2. Echocardiographic Analysis

**Figure 3:**
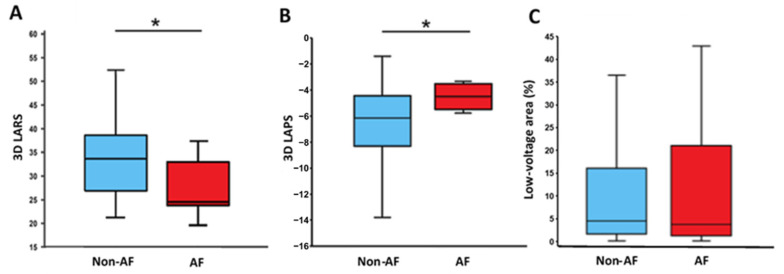
Left atrial reservoir strain (LARS, panel (**A**)), left atrial pump strain (LAPS, panel (**B**)), low-voltage area (LVA, panel (**C**)), and recurrence of atrial fibrillation (AF). 3D, three-dimensional; AF, patients with recurrence of atrial fibrillation; and Non-AF, patients without recurrence of atrial fibrillation. Asterisk denotes a significant difference (*p* < 0.05).

LV volume was normal in all, LVEF normal in most, and LVGLS reduced in all the patients. The LA was dilated in most patients, with 71 (76.3%) patients exhibiting a LAVI > 34 mL/m^2^ and 51 (54.8%) patients > 40 mL/m^2^. There was no difference in any of the parameters between patients with and without AF including LVGLS, 2D LARS, and 2D LAPS (Table 2). In contrast, 3D LARS and 3D LAPS were lower in patients with recurrent AF (*p* = 0.008 and *p* = 0.009, respectively; Table 2; Figure 3A,B).

### 7.3. LA Voltage Mapping

The number of LA mapping points during catheter intervention reached a median value of 1331 [882.3–2449.8]. LVA were present in 75 patients (81.5%) and the scar tissue covered a median of 4.4 [1.5–16.4]% of the LA wall (Table 2). There was no difference in LVA between the study groups (Non-AF-Group 4.4 [1.5 to 15.9]% vs. AF-Group 5.0 [1.5 to 21.5]%; *p* = 0.710; Figure 3C). No correlation between LVA and AF recurrence was observed.

### 7.4. Association with AF Recurrence

Over a median follow-up duration of 95 [83–110] days (mean = 110 days), AF could not be detected in 71 patients (87.1%; Non-AF-Group), while it was documented in 12 patients (12.9%; AF-Group). Paroxysmal AF had been observed in 61, persistent AF in 30, and long-standing AF in 2 patients before PVI, and AF recurred in 9, 3, and 0 patients, respectively.

Univariable Cox regression revealed that 3D LARS (HR 0.89 [0.80–0.98]; *p* = 0.025) and 3D LAPS (HR 1.40 [1.01–1.92]; *p* = 0.040) were the only parameters exhibiting a significant association with an increased risk of AF recurrence. All other parameters, including 2D LARS and 2D LAPS, as well as LVA, were not associated (Table 3). Univariable logistic regression yielded a very similar pattern of results.

### 7.5. Incremental Value of 3D LAS Parameters

In multivariable logistic regression, association of 3D LARS and 3D LAPS with an increased risk of AF recurrence during follow-up was independent of both the clinical model (including age, BMI, hypertension, and diabetes, Table 4) and the echocardiography model (including LVEF, LVEDVI, and biplane LAVI, Table 5). None of the other parameters examined showed a significant independence of the two models (Table 4 and Table 5).

While all the parameters improved the fitness of the models (X^2^), only 3D LARS and 3D LAPS induced a significant increase in the fitness of the nested echocardiography model (echocardiography model: X^2^ 6.97, *p* = 0.073; + 3D LARS X^2^ 11.98, *p* = 0.018; + 3D LAPS X^2^ 12.41, *p* = 0.015; Table 5). Again, this effect was not observed with any of the other clinical or echocardiographic parameters tested, including 2D LARS and 2D LAPS, as well as the 3D volume indices 3D LAVIR and 3D LAVIP.

### 7.6. Prediction of the AF Recurrence Probability 

Receiver operating characteristics (ROC) curve analysis showed that a 3D LARS < 26.5% (i.e., more impaired) predicted AF recurrence with a sensitivity of 66% and a specificity of 82% (Youden index 0.48, AUC 0.74 [95%CI 0.64–0.85], SE 0.054, *p* < 0.001). Similar observations were made with 3D LAPS ≤ 5.9% (Sens. 100%, Spec. 57%, Youden index 0.57, AUC 0.73 [95%CI 0.63–0.82], SE 0.077, *p* < 0.001). Comparison of the ROC curves for 3D LARS vs. 3D LAPS did not yield a significant difference between their AUC (*p* = 0.996)

**Figure 4:**
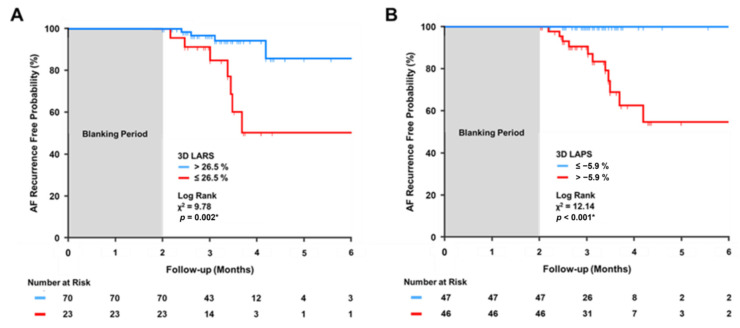
Kaplan–Meier curves for AF recurrence free probability. AF, atrial fibrillation; 3D, three-dimensional; X^2^, chi-square of Log Rank test; LARS, left atrial reservoir strain (LARS, panel (**A**)); and left atrial pump strain (LAPS, panel (**B**)). Asterisk denotes a significant Log Rank test (*p* < 0.05).

When these cut-off values were used for Kaplan–Meier analyses, patients with 3D LARS ≤ 26.5% or 3D LAPS > −5.9% showed a significantly higher probability of AF recurrence during the first six month of follow-up after the blanking period (Log Rank X^2^ = 9.78, *p* = 0.002 and X^2^ = 12.14, *p* < 0.001, Figure 4A and Figure 4B, respectively). 

## 8. Discussion

This prospective study demonstrates that impaired 3D LAS is associated with recurrence of AF in a real-world patient cohort undergoing PVI. Neither 3D volume indices nor 2D LAS or LA voltage mapping were associated with recurrence of AF under study conditions. This observation has potential implications for selection of patients undergoing PVI.

Atrial deformation was assessed in a prospective and standardized manner and determined in a 3D data set. The 3D method is free from geometric assumptions, measures deformation in all atrial segments (Table S1), and may reduce errors related to volume calculation and event timing known to occur with the 2D approach [25]. In the current dataset, the 3D method exhibited a good inter-observer and intra-observer reproducibility. The 3D LAS was lower in patients with AF recurrence, associated with recurrence, and able to differentiate between individuals with and without recurrence, suggesting that 3D LAS may predict outcome after PVI. Measurement of atrial deformation allows to determine atrial reservoir, conduit, and pump function [25,26]. Diminished LAS was observed with 3D LARS and 3D LAPS, indicating that atrial compliance and atrial contraction are reduced in patients with recurrent AF. Dilated cardiac chambers exhibit lower deformation due to altered chamber geometry [27]. Although LA volume was above the upper normal limit in most patients, it did not differ significantly in those with and those without recurrent AF, suggesting that atrial volume cannot account for lower deformation in the former group. Consistent with this interpretation, LAVI, LAVIR (left atrial reservoir volume index), and LAVIP (left atrial pump volume index) were not associated with recurrence of AF. These observations underscore that LAS is a functional parameter useful for assessment of outcome in patients undergoing PVI. 

The association of echocardiographic 3D LA strain with AF recurrence has not been explored except for a study performed in 42 patients with paroxysmal AF. That study was limited by a low patient number, application of LV strain module for assessing LA deformation, analysis of less well accepted parameters such as atrial circumferential and area strain, missing association of atrial reservoir strain (LARS) with recurrent AF, and lack of any data on atrial pump strain (LAPS) [16]. Thus, it is difficult to compare those outcomes with this current study, which to the best of our knowledge is the first one determining the association of 3D LARS and 3D LAPS with recurrence of AF. The current data support the inclusion of 3D LAS in the echocardiographic evaluation of patients undergoing PVI, particularly because all the volume-derived parameters such as LAVI, LAVIR, and LAVIP were not useful for predicting recurrence of AF and might improve work-up of this increasingly important patient group. A recent study performed by cardiac magnetic resonance confirms this interpretation [28].

A meta-analysis including 686 patients described that 2D LARS was significantly associated with AF recurrence [29]. In this current dataset, 2D LARS was lower in patients with recurrent AF, but not significantly different from those without. Consistent with this finding, 2D LARS did not exhibit a significant association with recurrence of AF. The difference between the meta-analysis and this current study may be related to the lower patient number in the latter. Nevertheless, the clearly significant association of 3D LAS in the smaller population studied in this current work suggests that 3D LAS may offer advantages over the 2D approach due to its stronger association with recurrent AF. The well characterized methodological advantages of the 3D compared to the 2D approach are consistent with this interpretation [25].

Little is known about the correlation of LAS with EAVM. A study performed in 22 patients found a relatively good correlation of 2D LAS with LVA using various low-voltage cut-offs [30]. However, that study measured atrial conduit strain, which is of little functional importance, and this may account for the observation that the correlation of LAVI with LVA was better than that of LAS with LVA in that study. Another study in 42 patients found that patients with LVA exhibited lower 2D LARS, and there was a negative correlation of LVA with LARS [31]. That population consisted of patients with either paroxysmal or persistent AF, and a large proportion of the population was in AF during analysis. This current study did not reveal a significant correlation between LVA and LAS, which was neither the case for the 2D nor the 3D approach. Differences in the study population including patient number and AF classification may account for the discrepant findings, the patient number of this current study being larger than that of the other studies.

Apart from the study population, additional factors may affect the association of LVA with recurrence of AF. Only a minority of patients included in this current study exhibited fibrotic tissue covering more than 10% of the total atrial wall, which is consistent with an early stage of LA wall fibrosis development [32]. Furthermore, different mapping methods, lower number of mapping points, different cut-off values for LVA, and measurement of LVA as a discrete variable may contribute to diverse findings [4,5,33,34,35]. Voltage maps are modulated by variations in cycle length and the direction of wave front activation, and therefore may be influenced by the conditions prevailing during their acquisition [36]. A recent intriguing study observed that the degree of fibrosis did not differ in patients with various stages of AF compared to control individuals, and electrophysiological abnormalities did not correlate with any fibrosis marker [37]. Therefore, atrial wall fibrosis may not be the only factor driving electrophysiological alterations leading to atrial fibrillation, suggesting that it might be advantageous to determine atrial wall deformation rather than its electrophysiological properties for predicting recurrence of AF.

## 9. Limitations

A major limitation of this prospective study is its single-center study design. Although the patient number is in a reasonable range, higher numbers would certainly be preferable for a multi-variable analysis. The PVI were conducted by four electrophysiologists using different types of mapping catheters, and both factors might have introduced additional variability in the EAVM. In some patients, recurrence of AF might have been missed due to lack of symptoms and non-continuous monitoring for AF. Follow-up duration of 3 months could be a limitation as well; however, a previous study has shown that recurrence within 3 months is also predictive for late recurrence of AF [38]. Taken together, validation in a multi-center cohort with more stringent conditions for mapping protocols as well as event monitoring may be required to provide more robust evidence on the results and minimize possible bias.

## 10. Conclusions

The main finding of this paper is that 3D LARS and 3D LAPS were associated with recurrence of AF after PVI, while 2D LARS, 2D LAPS, and LVA obtained from EAVM were not. 3D LARS and 3D LAPS improved the fitness of multivariable clinical and echocardiographic models. Therefore, the data suggest that 3D LARS and 3D LAPS may be applied for outcome prediction in patients undergoing PVI. Hence, 3D LARS and 3D LAPS may contribute to the identification of appropriate candidates for PVI and the personalized therapy of these patients.

## Figures and Tables

**Table 1:** Baseline characteristics.

Parameters	All (*n* = 93)	Non-AF (*n* = 81)	AF (*n* = 12)	*p*-Value
Age, years, median (IQR)	63.9 (58.9–70.3)	63.9 (57.8–69.4)	65.2 (60.0–72.1)	0.536
Men (*n*, %)	75 (81)	66 (81)	9 (75)	0.599
BMI, kg/m^2^, median (IQR)	26.0 (24.1–29.4)	26.0 (24.2–29.3)	24.9 (23.2–30.3)	0.571
BSA, m^2^, median (IQR)	2.0 (1.9–2.2)	2.0 (1.9–2.2)	2.0 (1.8–2.1)	0.205
Diabetes (*n*, %)	12 (13)	9 (11)	3 (25)	0.183
Hypertension (*n*, %)	49 (53)	42 (52)	7 (58)	0.676
SBP, mmHg, median (IQR)	129.5 (119.0–144.0)	129.0 (119.0–143.5)	132.0 (123.3–144.5)	0.605
DBP, mmHg, median (IQR)	77.0 (70.0–84.0)	77.0 (70.0–84.0)	76.0 (61.3–83.8)	0.412
Hypertensive heart disease (*n*, %)	6 (7)	5 (6)	1 (8)	1.000
Dilated cardiomyopathy (*n*, %)	5 (5)	5 (6)	0 (0)	-
Hypertrophic cardiomyopathy (*n*, %)	3 (3)	3 (3)	0 (0)	-
Coronary artery disease (*n*, %)	15 (16)	13 (16)	2 (17)	1.000
Ischemic heart disease (*n*, %)	1 (1)	1 (1)	0 (0)	-
Valvular heart disease (*n*, %)	23 (25)	20 (25)	3 (25)	0.981
-Moderate VHD (*n*, %)	14 (15)	12 (15)	2 (17)	1.000
-Severe VHD (*n*, %)	2 (2)	1 (1.0)	1 (8)	-
Renal insufficiency (*n*, %)	19 (20)	15 (18)	4 (33)	0.237
Sleep apnea (*n*, %)	8 (9)	7 (9)	1 (8)	0.972
Paroxysmal AF (*n*, %)	61 (66)	52 (64)	9 (75)	1.000
Persistent AF (*n*, %)	30 (32)	27 (33)	3 (25)	1.000
Long-standing AF (*n*, %)	2 (2)	2 (3)	0 (0)	-
NT-proBNP, ng/L, median (IQR)	269.5 (134.0–873.0)	247.5 (124.5–825.5)	491.5 (214.5–1077.5)	0.285
Anti-arrhythmic drugs (*n*, %)	16 (17)	14 (17)	2 (17)	1.000
Beta-blockers (*n*, %)	71 (76)	63 (78)	8 (67)	0.401
Calcium channel blockers (*n*, %)	17 (18)	15 (19)	2 (17)	0.863
Digoxin (*n*, %)	0 (0)	0 (0)	0 (0)	1.000
Oral anticoagulants (*n*, %)	88 (95)	76 (94)	12 (100)	0.379

BMI, body mass index; BSA, body surface area; SBP, systolic blood pressure; DBP, diastolic blood pressure; VHD, valvular heart disease; AF, atrial fibrillation; NT-pro BNP, *n*-terminal prohormone of brain natriuretic peptide.

**Table 2:** Echocardiographic and electroanatomical voltage map parameters.

Parameters, Median (IQR)	All (*n* = 93)	Non-AF (*n* = 81)	AF (*n* = 12)	*p*-Value
LVA, (cm^2^)	5.8 (2.0–23.8)	6.0 (2.0–21.0)	5.0 (2.3–47.5)	0.705
LVA, (%)	4.4 (1.5–16.4)	4.4 (1.5–15.9)	5.0 (1.5–21.5)	0.710
LAVI, (mL/m^2^)	42.0 (35.0–52.0)	41.0 (35.0–51.3)	52.5 (33.5–65.5)	0.176
3D LAVIR, (mL/m^2^)	45.0 (37.5–54.5)	43.5 (37.0–54.3)	54.2 (35.5–68.5)	0.199
3D LAVIP, (mL/m^2^)	16.9 (11.5–25.5)	16.0 (11.6–22.8)	22.4 (11.6–27.3)	0.294
2D LARS, (%)	42.1 (34.9–49.8)	43.6 (35.7–50.7)	39.6 (32.9–42.0)	0.083
2D LAPS, (%)	−17.9 (−20.4 to −13.5)	−18.0 (−21.0 to −14.0)	−16.6 (−18.8 to −11.6)	0.178
3D LARS, (%)	32.3 (26.6–38.1)	33.6 (27.0–38.6)	24.5 (23.8–33.0)	0.008 *
3D LAPS, (%)	−5.9 (−8.1 to −4.4)	−6.2 (−8.3 to −4.5)	−4.6 (−5.6 to −3.6)	0.009 *
LVEDVI, (mL/m^2^)	57.0 (45.5–68.5)	57.0 (47.3–69.0)	50.0 (43.5–57.0)	0.147
LVEF, (%)	55.0 (51.0–59.0)	55.0 (50.0–60.0)	56.0 (54.0–58.0)	0.517
LVGLS, (%)	−11.6 (−14.5 to −9.5)	−11.9 (−14.6 to −9.5)	−11.3 (−12.7 to −9.6)	0.421
LV Diastolic dysfunction (*n*, %)	7.0 (7.5)	6.0 (7.4)	1.0 (8.3)	0.920
RAESA, (cm^2^)	22.5 (19.0–25.0)	21.5 (18.3–25.0)	24.0 (19.0–27.5)	0.518
RVEDAI (cm^2^/m^2^)	10.6 (9.1–11.8)	10.6 (9.1–11.9)	10.2 (8.6–11.3)	0.500
FAC (%)	38.0 (35.0–41.0)	38.0 (35.0–41.0)	39.0 (37.5–41.5)	0.527
TAPSE (mm)	19.0 (15.0–23.0)	19.0 (15.0–23.0)	19.0 (15.5–23.5)	0.941

Asterisk denotes a significant difference (*p* < 0.05). IQR, interquartile range; LVA, low-voltage area; LAVI, left atrial volume index; 3D, three-dimensional; LAVIR, left atrial reservoir volume index; LAVIP, left atrial pump volumeindex; 2D, two-dimensional; LARS, left atrial reservoir strain; LAPS, left atrial pump strain; LVEDVI, left ventricular end-diastolic volume index; LVEF, left ventricular ejection fraction; LVGLS, left ventricular global longitudinal strain; RAESA, right atrial end-systolic area; RVEDAI, right ventricular end-diastolic area index; FAC, fractional area change; and TAPSE, tricuspid annular plane systolic excursion.

**Table 3:** Univariable Cox regression analysis for atrial fibrillation recurrence.

Variables	Cox Regression			Model Fitness	
	HR	95%CI	*p*-Value	X^2^	X^2^ *p*-Value
Diabetes	1.87	0.48–7.35	0.368	0.73	0.393
Hypertension	1.43	045–4.53	0.541	0.38	0.378
Renal insufficiency	1.99	0.58–6.82	0.274	1.10	0.295
Sleep apnea	0.37	0.05–3.06	0.358	1.08	0.300
NT-proBNP (ng/L)	1.00	1.00–1.00	0.860	0.03	0.862
LVEDVI (mL/m^2^)	0.95	0.90–1.00	0.041 *	4.85	0.028 *
LVEF (%)	1.00	0.94–1.07	0.996	0.00	0.994
LVGLS (%)	1.08	0.90–1.30	0.395	0.74	0.391
LAVI (mL/m^2^)	1.02	0.99–1.05	0.233	1.27	0.260
3D LAVIR (mL/m^2^)	1.01	0.98–1.05	0.287	1.16	0.297
3D LAVIP (mL/m^2^)	1.05	0.98–1.13	0.203	1.59	0.207
2D LARS (%)	0.95	0.90–1.00	0.556	3.90	0.049 *
2D LAPS (%)	1.10	1.00–1.20	0.195	1.70	0.187
3D LARS (%)	0.89	0.81–0.99	0.025 *	6.48	0.011 *
3D LAPS (%)	1.40	1.02–1.92	0.040 *	7.04	0.008 *
LVA (cm^2^)	1.00	0.98–1.02	0.947	0.01	0.946
LVA (%)	0.51	0.00–64.62	0.787	0.08	0.784

Asterisk denotes a significant difference (*p* < 0.05). HR, hazard ratio; CI, confidence interval; NT-pro BNP, *n*-terminal prohormone of brain natriuretic peptide; LVEDVI, left ventricular end-diastolic volume index; LVEF, left ventricular ejection fraction; LVGLS, left ventricular global longitudinal strain; LAVI, left atrial volume index; 3D, three-dimensional; LAVIR, left atrial reservoir volume index; LAVIP, left atrial pump volume index; 2D, two-dimensional; LARS, left atrial reservoir strain; LAPS, left atrial pump strain; and LVA, low-voltage area.

**Table 4:** Multivariable logistic regression analysis for atrial fibrillation recurrence (clinical model).

Variables	Multivariable Logistic Regression			Model Fitness	
	OR	95%CI	*p*-Value	X^2^	X^2^ *p*-Value
Age (years)	1.03	0.95–1.11	0.514	2.81	0.590
BMI, (kg/m^2^)	0.94	0.83–1.06	0.323		
Hypertension	1.14	0.29–4.43	0.850		
Diabetes	3.42	0.62–18.89	0.159		
+3D LAVIR (mL/m^2^)	1.04	0.98–1.08	0.127	5.05	0.442
+3D LAVIP (mL/m^2^)	1.05	0.99–1.07	0.106	5.86	0.398
+2D LARS (%)	0.90	0.86–1.01	0.076	6.10	0.299
+2D LAPS (%)	1.10	0.96–1.26	0.157	4.60	0.461
+3D LARS (%)	0.88	0.79–0.98	0.025 *	9.23	0.100
+3D LAPS (%)	1.55	1.08–2.22	0.019 *	10.43	0.064
+LVA (%)	1.00	0.95–1.06	0.907	2.82	0.728

Asterisk denotes a significant difference (*p* < 0.05). HR, hazard ratio; CI, confidence interval; BMI, body mass index; 3D, three-dimensional; LAVIR, left atrial reservoir volume index; LAVIP, left atrial pump volume index; 2D, two-dimensional; LARS, left atrial reservoir strain; LAPS, left atrial pump strain; and LVA, low-voltage area.

**Table 5:** Multivariable logistic regression analysis for atrial fibrillation recurrence (echocardiographic model).

Variables	Multivariable Logistic Regression			Model Fitness	
	OR	95%CI	*p*-Value	X^2^	X^2^ *p*-Value
LVEF (%)	1.01	0.93–1.10	0.892	6.97	0.073
LVEDVI (mL/m^2^)	0.96	0.91–1.01	0.082		
LAVI (mL/m^2^)	1.04	1.01–1.09	0.028 *		
+3D LAVIR (mL/m^2^)	1.04	0.99–1.11	0.095	7.21	0.241
+3D LAVIP (mL/m^2^)	1.01	0.98–1.10	0.094	8.03	0.156
+2D LARS (%)	0.90	0.88–1.03	0.200	8.77	0.057
+2D LAPS (%)	1.10	0.95–1.26	0.216	8.94	0.062
+3D LARS (%)	0.88	0.79–0.99	0.040 *	11.98	0.018 *
+3D LAPS (%)	1.45	1.00–2.09	0.048 *	12.41	0.015 *
+LVA (%)	0.99	0.94–1.05	0.762	7.12	0.130

Asterisk denotes a significant difference (*p* < 0.05). LVEF, left ventricular ejection fraction; LVEDVI, left ventricular end diastolic volume index; LAVI, left atrial volume index; 3D, three-dimensional; LAVIR, left atrial reservoir volume index; LAVIP, left atrial pump volume index; 2D, two-dimensional; LARS, left atrial reservoir strain; LAPS, left atrial pump strain; and LVA, low-voltage area.

## Data Availability

The data underlying this article will be shared on reasonable request to the corresponding author.

## Supplementary Materials

The following supporting information can be downloaded at: https://www.mdpi.com/article/10.3390/jcm12113696/s1, Table S1: 3-dimensiontal left atrial strain in segmented areas.
