# Supplementary material for: 3D Atrial Strain for Predicting Recurrence of Atrial Fibrillation after Pulmonary Vein Isolation
Source: J Clin Med. 2023 May 26;12(11):3696. doi: 10.3390/jcm12113696 (PMC10253337; doi:10.3390/jcm12113696)
Supplement: Supplementary file 1 [file jcm-12-03696-s001.zip › jcm-2356916-supplementary.pdf]

Supplemental Table S1: 3-dimensiontal left atrial strain in segmented areas.

Parameters [%], median [IQR]		All	Non-AF	AF	p-value
**Anterior**	LARS	33.3 [29.9-37.1]	33.4 [27.0-38.4]	24.1 [22.1-34.2]	0.012*
	LAPS	-4.4 [-5.6 to -3.6]	-4.8 [-5.8 to -3.7]	-3.8 [-4.3 to -3.5]	0.018*
**Posterior**	LARS	31.7 [25.2-39.8]	32.3 [26.3-40.4]	24.7 [21.8-35.4]	0.019*
	LAPS	-5.8 [-7.8 to -3.7]	-6.4 [-8.0 to -3.8]	-4.6 [-5.7 to -2.9]	0.019*
**Septal**	LARS	28.8 [23.8-34.4]	30.4 [24.7-36.1]	22.7 [0.5-28.1]	0.002*
	LAPS	-5.7 [-8.7 to -3.8]	-6.1 [-8.9 to -3.9]	-4.4 [-5.5 to -3.5]	0.017*
**Lateral**	LARS	34.7 [30.0-39.4]	35.3 [30.3-39.6]	30.3 [29.5-31.9]	0.033*
	LAPS	-7.5 [-10.7 to -5.0]	-7.9 [-11.7 to -5.3]	-5.2 [-7.1 to -4.3]	0.010*
**Roof**	LARS	32.5 [26.0-39.1]	34.3 [26.6-39.8]	25.1 [23.7-30.9]	0.006*
	LAPS	-5.8 [-8.3 to -4.2]	-6.5 [-8.5 to -4.2]	-4.5 [-5.4 to -3.8]	0.019*

*P<0.05.
LARS, left atrial reservoir strain; LAPS, left atrial pump strain.
